# In-vivo detection of white adipose tissue browning: a multimodality imaging approach

**DOI:** 10.1038/s41598-023-42537-9

**Published:** 2023-09-19

**Authors:** Leah R. Holmes, John C. Garside, Jonathan Frank, Eric Livingston, Jonas Snyder, Nada Abu Khalaf, Hong Yuan, Rosa T. Branca

**Affiliations:** 1 Department of Physics and Astronomy, University of North Carolina at Chapel Hill, Chapel Hill, NC 27599 USA; 2 Biomedical Research Imaging Center, University of North Carolina at Chapel Hill, Chapel Hill, NC 27599 USA

**Keywords:** Imaging, X-ray tomography, Preclinical research

## Abstract

Detection and differentiation of brown fat in humans poses several challenges, as this tissue is sparse and often mixed with white adipose tissue. Non-invasive detection of beige fat represents an even greater challenge as this tissue is structurally and functionally more like white fat than brown fat. Here we used positron emission tomography with ^18^F-fluorodeoxyglucose, computed tomography, xenon-enhanced computed tomography, and dynamic contrast-enhanced ultrasound, to non-invasively detect functional and structural changes associated with the browning process of inguinal white fat, induced in mice by chronic stimulation with the β_3_-adrenergic receptor agonist CL-316243. These studies reveal a very heterogeneous increase in baseline tissue radiodensity and xenon-enhanced radiodensity, indicative of both an increase in adipocytes water and protein content as well as tissue perfusion, mostly in regions that showed enhanced norepinephrine-stimulated perfusion before CL-316243 treatment. No statistically significant increase in ^18^F-fluorodeoxyglucose uptake or norepinephrine-stimulated tissue perfusion were observed in the mice after the CL-316243 treatment. The increase in tissue-water content and perfusion, along with the negligible increase in the tissue glucose uptake and norepinephrine-stimulated perfusion deserve more attention, especially considering the potential metabolic role that this tissue may play in whole body metabolism.

## Introduction

In the past two decades it has become clear that adipose tissue comes in different “shades”^1^ and is responsible for more than just lipid storage^2–4^. White Adipose Tissue (WAT), which comprises most of the common adipose tissue in adult humans, is dispersed throughout the entire body and helps to store energy, protect against dermal infection, provide thermal insulation and cushion against mechanical stressors^2^. This tissue is also the largest endocrine tissue found in the body^3^. Brown Adipose Tissue (BAT), originally thought to be relevant only for temperature regulation, has recently been found to modulate both fatty acids and glucose blood levels^5–7^. In BAT, adipocytes contain many mitochondria in which fatty acids are oxidized to produce heat. Heat production in brown adipocytes is mediated by the action of the uncoupling protein 1 (UCP1), a protein found in the inner mitochondrial membrane that enables protons to leak back into the matrix without synthesizing adenosine triphosphate^8^. The UCP1 protein is activated by long chain fatty acids, which also represent the major substrate for heat production in BAT^9,10^. Unlike WAT, BAT is highly vascularized and, during stimulation of BAT thermogenesis, blood flow to BAT increases by several folds to efficiently redistribute the heat produced to other tissues^11–13^. While BAT activation is known to regulate blood glucose and fatty acids and to be associated with a healthier metabolic profile in humans, recent studies in mice have shown that browning of white adipose tissue, which leads to beige fat, either by gene editing of preadipocytes or autologous fat transplants, also improves glucose and lipid homeostasis^14–16^.

Compared to brown adipocytes*,* beige adipocytes are morphologically more like white adipocytes than brown adipocytes. They are also found interspersed within white adipocytes, making their detection even more challenging^17–19^. While there exist several imaging modalities that have been used for detecting and differentiating BAT from WAT^20,21^, there are fewer than have been used for the detection of beige fat in vivo*.* So far, positron emission tomography using the fluorodeoxyglucose ^18^F radiotracer in combination with computed tomography (^18^F-FDG-PET/CT) has been the most common tomographic imaging modality used for the detection of beige adipocytes in vivo*.* Previous studies have reported an increase in glucose uptake in the inguinal depot, the WAT depot that is more likely to brown in mice after chronic stimulation with cold or the selective β3-adrenergic receptor agonist CL-316243, although some results have been contrasting^22,23^. In addition to fluorodeoxyglucose, other radiopharmaceutical tracers have also been explored for the detection of beige fat, such as TPSO-18 kDa (TPSO), a marker for mitochondrial expression^24^. Compared to ^18^F-FDG, the advantage of this tracer is that uptake does not require tissue stimulation. Recently, multispectral optoacoustic tomography has also been used for the detection of beige fat in mice^25^. However, because the penetration depth of this technology is limited to 3–5 cm below the tissue surface, its human translation may pose some challenges.

Xenon-Enhanced Computed Tomography (XECT) and Dynamic Contrast-Enhanced Ultrasound (DCEUS) are imaging modalities that have been used for the detection of BAT^26–30^, but never for the detection of beige fat. In XECT one takes advantage of its radio-dense properties, which are similar to that of iodine, another common CT vasculature contrast agent. Because of its lipophilic properties this gas is also an ideal perfusion agent, especially for fatty tissues. We recently demonstrated that xenon gas, when used as a contrast agent in CT scans, enables detection and differentiation of the highly perfused BAT from WAT in CT images^26^. This was demonstrated not only in lean mice, which present a very hydrated and perfused BAT, but also in obese mice in which the main interscapular BAT morphologically resembles beige fat. More recently, this technique was used to detect small clusters of BAT located within the supraclavicular, axillary, and perirenal regions of lean non-human primates and non-human primates with obesity^27^.

Contrast enhanced ultrasound (CEUS) is a technique that uses air-filled microbubbles, a vasculature contrast agent that efficiently scatters ultrasound waves, to enhance the vascular contribution of tissues to the ultrasound signal. The vasculature signal enhancement obtained after microbubble injection can been used to differentiate the highly vascularized interscapular BAT from the less vascularized WAT, especially during norepinephrine stimulation of thermogenesis, when the local increase in tissue perfusion leads to an increase in the microbubble/tissue ratio and to a selective enhancement of the BAT ultrasound signal. Alternatively, from the analysis of the time-intensity curve (Dynamic-CEUS or DCEUS) one can extract relevant tissue parameters such as tissue blood volume and blood flow^31,32^. This type of analysis has been used to identify and quantify the interscapular BAT depot in rodents^28,29^ and the supraclavicular BAT depot in humans^33^, as well as to detect a reduced response to norepinephrine-induced interscapular BAT blood flow in UCP1^−/−^ mice^29^, which has not been detected by XECT^34^ or high resolution laser doppler imaging^35^.

The scope of this study was to evaluate the ability of these three clinically relevant imaging modalities, ^18^F-FDG-PET, XECT, and DCEUS, to detect structural and functional changes that occur in mice in inguinal white fat during the browning process *in-vivo*.

## Results

### ^18^F-FDG-PET results

**Figure 1:**
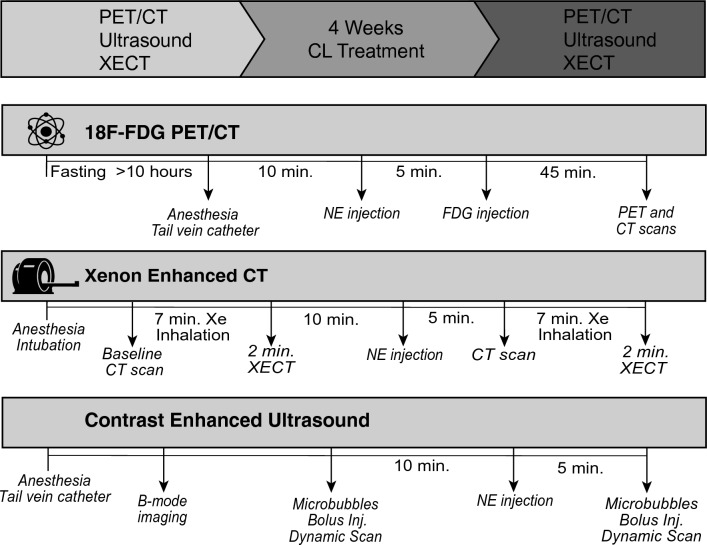
Overview of study design. PET (Positron Emission Tomography), CT, and US studies were conducted in the same animals on three different days, both before and after a 4-week treatment with the β3-adrenergic agonists CL-316243, injected intraperitonellay at a dose of 1 mg/kg. For the ^18^F-FDG-PET/CT studies, mice were fasted overnight for more than 10 h. Norepinephrine-stimulated tissue glucose uptake was then quantified in the inguinal depot, before and after CL-316243 treatment. CT, xenon-enhanced CT, and dynamic contrast-ultrasound scans were performed before and after norepinephrine injection during the same imaging session, both before and after CL-316243 treatment.

**Figure 2:**
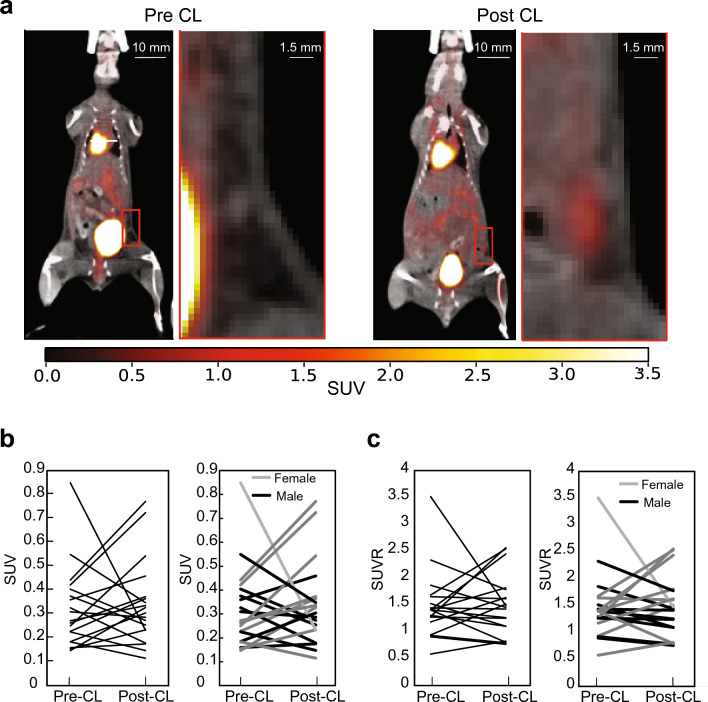
(**a**) ^18^F-FDG-PET/CT coronal fusion images of the same mouse acquired pre- and post-CL-316243 treatment. A zoom in of the inguinal area (red box) shows a localized increase in glucose uptake in the inguinal fat area; (**b**) Plots showing a non-statistically significant (p > 0.05, paired t-test) change in standardized uptake values (SUV) measured in the mice pre and post CL-316243 treatment. The plot on the right includes all mice, and the one on the left separates male from females; (**c**) Plots showing a non-statistically significant (p > 0.05, paired t-test) change in standardized uptake value ratios (SUVR) obtained in the mice pre and post CL-316243 treatment. The plot on the left includes all mice while the one on the right present males and females in black and gray colors, respectively.

Figure 1 shows an overview of the timeline of this study and of the three imaging protocols used in this study. Figure 2 shows an example of the ^18^F-FDG-PET/CT images acquired pre and post CL-316243 treatment. For quantification, ^18^F-FDG-uptake was measured in each mouse by using P-MOD-4 software (PMOD Technologies Ltd., Zurich, Switzerland) and Horos software (https://horosproject.org/). Volumes of interest (VOIs) were drawn by two independent observers in the most hyperintense region located within the inguinal fat depot, first in the post-CL-316243 scans and then in the same region for the pre-CL-316243 scans. It is important to note that a consistent HU threshold could not be used to differentiate beige fat from nearby white fat as the increase in tissue radiodensity observed in post-CL-316243 CT scans (in most cases less than 20 HU) was comparable to the difference in inguinal radiodensity observed across the animals. To normalize for differences in body weight, administration dose and tracer plasma clearance, the inguinal SUV (Standardized Uptake Value) was normalized using the SUV in the triceps to obtain the SUV ratios (SUVR). All values obtained are reported in Tables 1–3 in the Supplementary Material, along with the associated Kendall-tau rank test to assess the agreement between the two readers.

As shown in Fig. 2, a variable increase in glucose uptake, averaged across two observers, was detected in the mice post-CL-316243 treatment. The enhancement in glucose uptake varied widely with some mice showing an increase and some showing a decrease in SUVR.

### Xenon enhanced CT results

**Figure 3:**
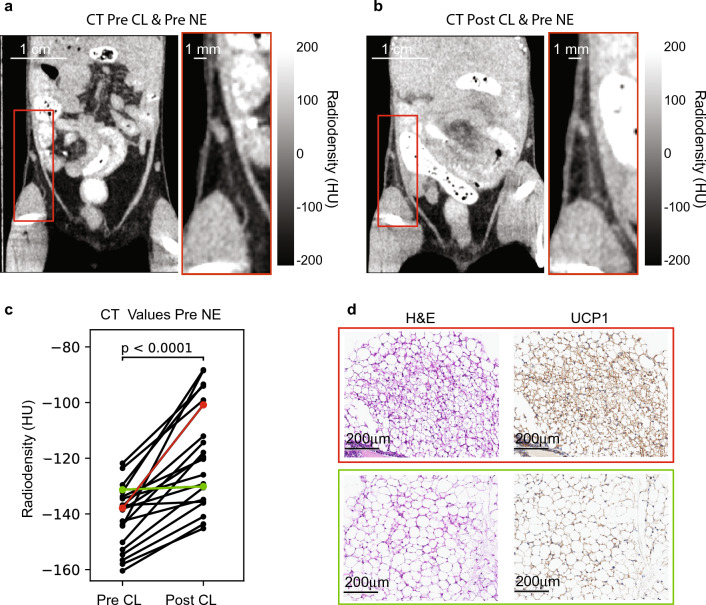
(**a**) CT coronal images acquired from a mouse before CL-316243 treatment showing the inguinal white fat depot adjacent to the inguinal lymph node and the external iliac artery; (**b**) CT coronal image acquired from the same mouse after CL-316243 treatment, showing an increase in tissue radiodensity near the external iliac artery; (**c**) Inguinal tissue radiodensity as measured in each mouse before and after CL-316243 treatment. A paired t-test shows a significant (p < 0.0001) increase in tissue radiodensity post CL-316243 treatment; (**d**) Hematoxylin and eosin staining and immunostaining of UCP1 expression for one mouse that showed significant increase in tissue radiodensity (orange line) and one that did not (green line).

All CT images were analyzed by using ITK-snap (http://www.itksnap.org/pmwiki/pmwiki.php) and Horos software by two different investigators. For these studies, the Hounsfield values for the pre and post CL-316243 scans were first calibrated by using a water/air phantom. For the analysis, beige tissue was identified first in post-CL-316243 scans in the area adjacent to the external iliac artery near the inguinal lymph node. The same region was then identified in the pre-CL-316243 scans (Fig. 3a,b). All radiodensity values obtained by the two independent readers for this study are reported in Tables 4, 5 in the Supplementary Material, along with its associated Kendall tau rank test.

**Figure 4:**
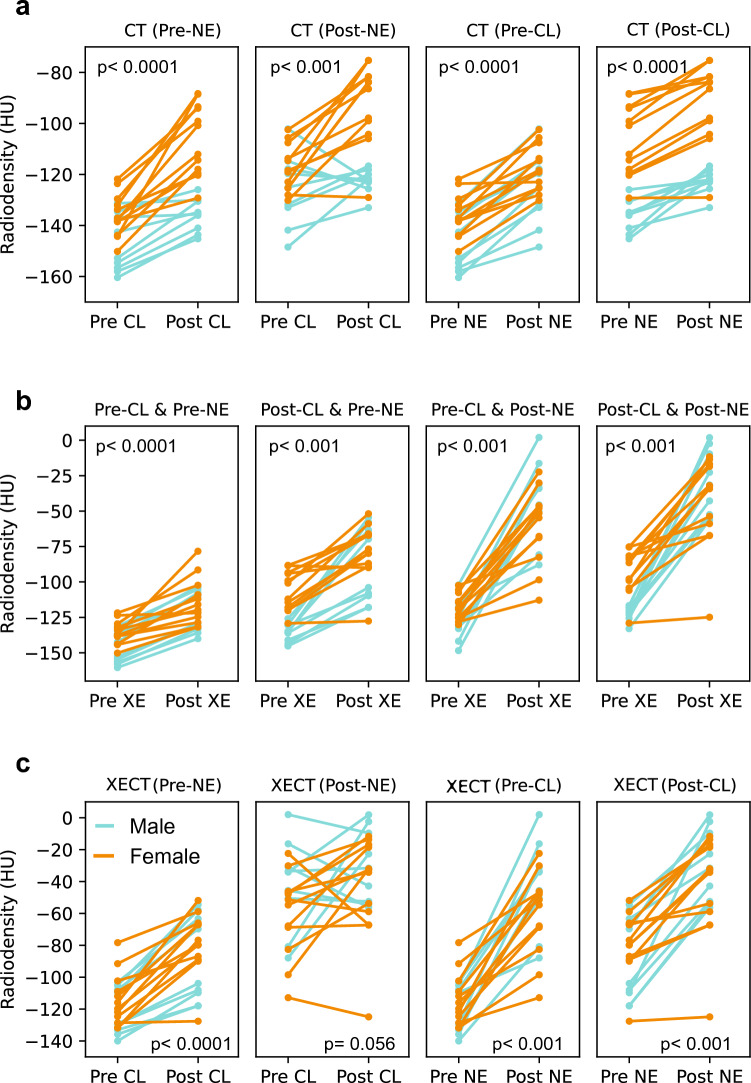
Mean tissue radiodensities obtained from all mice analyzed (p-values are reported for combined male and female mouse data): (**a**) Mean CT inguinal tissue radiodensities. An increase in CT tissue radiodensity is observed after CL-316243 treatment in all mice. An increase in tissue radiodensity is also observed after NE injection, both in pre and post CL-316243 scans; (**b**) Mean tissue radiodensities obtained before and after a 7-min xenon inhalation. In all mice tissue radiodensity increased except in one mouse, where tissue radiodensity did not increase after xenon inhalation; (**c**) Tissue radiodensity values as obtained after xenon inhalation, before and after CL-316243 treatment, and before and after norepinephrine injection. Baseline tissue radiodensity after xenon inhalation increased post CL-316243 treatment, indicating an increase in tissue perfusion. No consistent increase in tissue radiodensity was observed in post-NE scans. In all cases, tissue radiodensity increased post norepinephrine injection, indicating an increase in tissue perfusion after NE.

A statistically significant enhancement in tissue radiodensity ($22\pm 14 HU,2P\left(T\le t\right)<0.0001$) was observed in the mice after CL-316243 treatment (Fig. 3c). The observed increase in radiodensity indicated an overall increase in tissue water and protein content due either to an increase in tissue vascularization and/or to the conversion of white adipocytes into beige adipocytes. The latter was confirmed histologically by the presence of small, UCP1-positive, multilocular adipocytes in the region (Figs. 3d and Fig. S1). The increase was statistically higher (2-sides heteroscedastic t-test p value of < 0.0001 with Cohen’s d value of 1.27) in female ($30\pm 13 HU,2P\left(T\le t\right)<0.0001)$ than in male ($12\pm 7 HU,2P\left(T\le t\right)=0.0015$) mice. A statistically significant increase in tissue radiodensity was also observed when comparing non-enhanced CT images obtained after norepinephrine injection to those obtained before norepinephrine injection, both before ($20\pm 8 HU, 2P\left(T\le t\right)<0.0001$ and after ($14\pm 7.4HU, 2P\left(T\le t\right)<0.0001)$ CL-316243 treatment (Fig. 4). In this case no statistically significant differences were noted between the enhancement observed in male and female (2-sides heteroscedastic t-test p value > 0.05).

**Figure 5:**
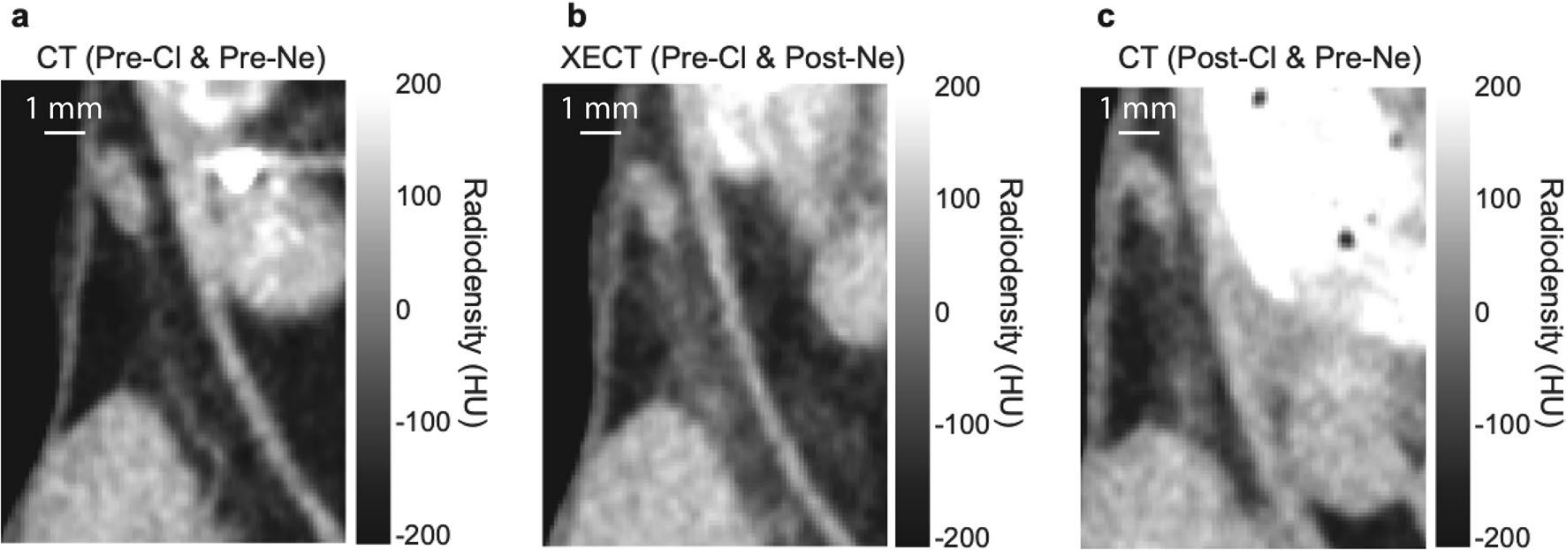
Selective radiodensity enhancement in region near the external iliac artery observed both before CL-316243 treatment in XECT scan and after CL-316243 treatment in CT scans: (**a**) A magnified view of the inguinal fat near the right inguinal lymph node for one of the mice showing non enhanced CT images acquired before CL-316243 treatment, before NE injection and before xenon inhalation; (**b**) A magnified view of the same mouse acquired before CL-316243 treatment, after NE injection and after 7-min xenon inhalation showing a selective enhancement of the fat area adjacent to the external iliac artery; (**c**) A magnified view of an image acquired on the same mouse after CL-316243 treatment, before NE injection and before xenon inhalation showing also a selective enhancement of the fat area adjacent to the external iliac artery.

Increase in tissue radiodensity was also observed in all mice right after the 7-min xenon inhalation (Fig. 4b). Specifically, an average increase of $$24\pm 12 HU$$ and $$36\pm 22 HU$$ was observed due to xenon inhalation before norepinephrine injection in pre-CL-316243 (PreCL&PreNE plot) and post-CL-316243 scans (PostCL&PreNE plot), respectively. These values were statistically different ($${t}_{c}=2, 2P\left(T\le t\right) = 0.005)$$. After norepinephrine injection the increase in tissue radiodensity after a 7-min xenon inhalation was even higher (Fig. 4b PreCL&PostNE plot), with an average value of $$69\pm 29 HU$$ and $$67\pm 33 HU$$ before (Fig. 4b PreCL &PostNE plot) and after (Fig. 4b PostCL&PostNE plot) CL-316243 treatment, respectively. These values were significantly greater ($$p<0.0001)$$ than those obtained pre-norepinephrine injection, but not significantly different from each other ($$p=0.8)$$. Interestingly, the increase in tissue radiodensity observed after CL-316243 treatment occurred primarily in the region next to the external iliac artery, a region that also showed, in xenon enhanced CT scans, selective radiodensity enhancement after norepinephrine injection even before CL-316243 treatment (Fig. 5).

### Dynamic contrast ultrasound results

**Figure 6:**
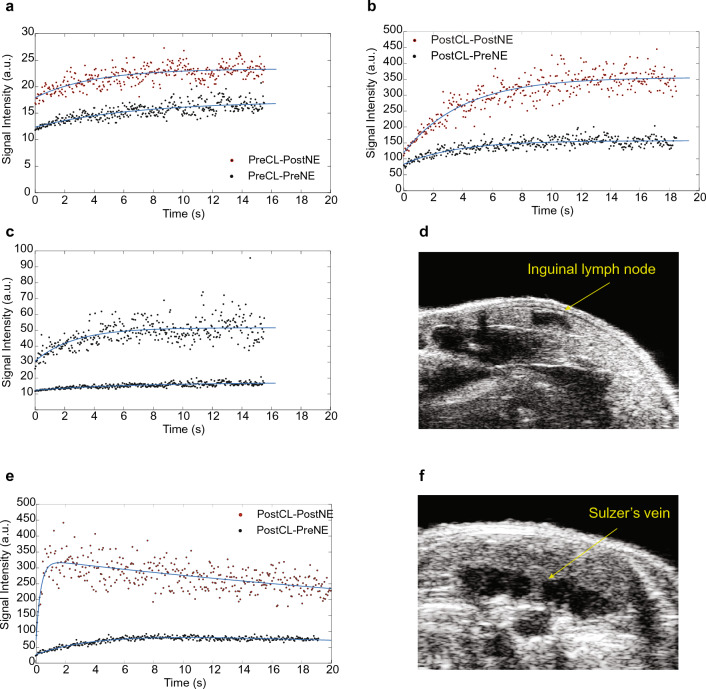
Acoustic signal intensity, as function of time, collected over 18 s after microbubble destruction in the inguinal fat depot and in the interscapular BAT of the same mouse. (**a**) Acoustic signal intensity acquired before CL-316243 treatment from one of the mice showing an increase in signal intensity after NE injection. Fitting parameters were A = 5.0 $\pm$ 0.5 a.u., and $\beta$ = 0.15 $\pm$ 0.04 s^−1^ before NE injection (adjusted R-square 0.60), and A = 5.5 $\pm$ 0.5 a.u., and $\beta$ = 0.25 $\pm$ 0.07 s^−1^ (adjusted R-square 0.52) after NE injection; (**b**) Acoustic signal intensity acquired after CL-316243 treatment from the same mouse also showing an increase in signal intensity after NE injection. Fitting parameters were A = 74 $\pm$ 5 a.u., and $\beta$ = 0.28 $\pm$ 0.05 s^−1^ before NE injection (adjusted R-square 0.66), and A = 237 $\pm$ 13 a.u., and $\beta$ = 0.25 $\pm$ 0.03 s^−1^ (adjusted R-square 0.8) after NE injection; (**c**) Acoustic signal intensity acquired before CL-316243 treatment and before NE injection from two adjacent regions. Fitting parameters were A = 21.0 $\pm$ 2 a.u., and $\beta$ = 0.38 $\pm$ 0.1 s^−1^ (adjusted R-square 0.41) and A = 5.0 $\pm$ 0.5 a.u., and $\beta$ = 0.15 $\pm$ 0.04 s^−1^ (adjusted R-square 0.60); (**d**) B-mode image showing the inguinal lymph-node used as a landmark; (**e**) Acoustic signal acquired from the interscapular brown fat of the same mouse displaying a remarkable increase in tissue perfusion after norepinephrine injection. Fitting values are A = 82 $\pm$ 15 a.u., and $\beta$ = 0.2 $\pm$ 0.05 s^−1^ before NE injection (adjusted R-square 0.86), and A = 250 $\pm$ 50 a.u., and $\beta$ = 3 $\pm$ 1 s^−1^ after NE injection (adjusted R-square 0.4); In this case, in order to account for the finite half-life of microbubbles in circulation, the data were fit using the equation *S(t)* = *A* × *(1 − e*^*−βt*^*) e*^*ct*^*;* (**f**) B-mode image of the interscapular BAT showing the Sulzer’s vein, the main vein that drains the interscapular BAT depot.

Dynamic ultrasound data was acquired from the inguinal depot of mice before and after norepinephrine injection and before and after CL-316243 treatment (n = 17). Figure 6 shows an example of the dynamic signal intensity curves acquired from the inguinal region of one of the mice, along with those acquired from the interscapular brown fat of the same mouse on a different day, for reference. Additional examples of dynamic ultrasound data curves acquired in this study are presented in Fig. S3 of the Supplementary Material along with the result of the dynamic curve fitting, used to extrapolate tissue blood flow and blood volume (Table 7-Supplementary Material). Both curves show an increase in tissue perfusion after norepinephrine injection, consistently with CT and xenon-enhanced CT results. However, this increase was not observed in all mice and was significantly smaller than the increase observed in the interscapular brown fat depot by tenfold. Additionally, consistently with CT results, the inguinal region appeared highly heterogenous, with nearby regions showing different dynamic curve profiles.

## Discussion

In this study we evaluated three imaging modalities that have been previously used for the detection of brown adipose tissue in humans or non-human primates on their ability to detect browning of white fat *in-vivo* in mice. Browning of white adipose tissue has been proposed to increase metabolic rate and regulate blood glucose level. In mice, it can be induced by either chronic cold exposure or, as done here, by chronic β_3_-adrenergic stimulation.

Consistent with previously reported studies in mice, a 4-week treatment with the β_3_-adrenergic receptor agonist CL-316243 induced browning of white fat in the inguinal fat depot of mice. In our studies, the browning process was detected first in CT scans, which showed a small but significant increase in the radiodensity of this tissue after CL-316243 treatment. After 4 weeks of CL-316243 treatment, the increase in tissue radiodensity was small and highly heterogenous, with regions adjacent to the external iliac artery showing the highest radiodensity increase. This increase was indicative of either an increase in tissue vascularization and/or cell water and protein content, expected with the conversion of unilocular adipocytes into multilocular UCP1-positive adipocytes, whose presence was confirmed histologically post-mortem.

A small radiodensity increase was also observed in the same tissue after norepinephrine injection, both before and after CL-316243 treatment. Because this increase, observed more than 15 min after the last xenon inhalation, was comparable in size to the increase in tissue radiodensity observed right after the first xenon inhalation, it could not be attributed to residual xenon in the tissue. Also, because it was observed only after 5 min from the norepinephrine injection and it was comparable in size to the increase observed after the 4-week treatment with CL-316243, it could not be attributed to the expected reduction in tissue fat content either and had, instead, to be attributed to an increase in tissue perfusion following norepinephrine injection.

Inhalation of xenon led to an increase in the baseline tissue radiodensity, both before and after CL-316243 treatment. The additional increase in tissue radiodensity observed after xenon inhalation was higher in post CL-316243 scans than in pre CL-316243 scans. This indicated that the conversion of unilocular adipocytes into multilocular adipocytes, which alone would have led to a reduction in the solubility of xenon and its induced radiodensity enhancement, was concurrent with an increase in tissue baseline perfusion.

The xenon-induced radiodensity increase was also significantly higher after norepinephrine injection than before norepinephrine injection in both pre-CL-316243 scans and post-CL-316243 scans. The additional tissue radiodensity enhancement observed in xenon-enhanced CT scans right after norepinephrine injection is indicative of an increase in tissue perfusion during stimulation of non-shivering thermogenesis after norepinephrine injection, consistently with the smaller increase in radiodensity also seen in non-enhanced CT scans. The small increase in tissue radiodensity seen in non-enhanced CT scans after norepinephrine injection is essentially amplified in xenon-enhanced CT scans as the increase in tissue blood flow is expected to reduce xenon wash-in time and increase xenon tissue uptake. As previously shown, increase in blood flow after norepinephrine injection is, although not indicative of BAT thermogenesis^35^, a hallmark of brown adipose tissue, both in rodents^13,36^ and in humans^37^. Interestingly, this increase occurred not just after browning induction, but also before browning induction, indicating that inguinal fat is different than other white fat depots, even before browning occurs. In pre-CL-316243 scans this increase led to a selective uptake of xenon in the same regions that later underwent the highest increase in tissue radiodensity. These findings suggest that, within the inguinal region, regions that are capable of experience the highest vasculature response to norepinephrine stimulation before browning induction are also the ones that are more likely to undergo browning. Given the possible role of sympathetic system dysfunction in obesity, these findings deserve further attention.

While we also found a slight increase in xenon uptake in pre-norepinephrine scans after CL-316243 treatment, which indicates an increase in tissue perfusion, we did not find an increase in xenon uptake in post-norepinephrine scans after CL-316243 treatment. This suggest that the tissue vasculature response to norepinephrine was not altered by chronic exposure to CL-316243.

Consistently with previous studies^22^, we did not measure a statistically significant increase in norepinephrine stimulated glucose uptake in inguinal fat. These results agree with those previously reported by Labbe’ et al., after chronic cold or CL-316243 treatment in male C57Bl/6 mice, which showed lack of statistically significant increase in beige fat tissue metabolism after CL-316243 treatment^22^. The low/absent increase in glucose uptake after CL-316243 treatment deserves more attention, especially considering the concurrent increase in tissue perfusion following norepinephrine injection and the increase in tissue water/protein content observed after CL-316243 treatment. A possible explanation could be the relatively low uptake in this tissue, even after CL-316243 treatment, and the low resolution of the PET scan (1 mm at the centre of the detector and ~ 2 mm in the inguinal region) which, along with the relatively small size of the inguinal depot (~ 100 mm^3^) and the heterogeneity of the browning process in the region, may have led to partial volume effects.

When we looked at potential correlations between the increase in CT image radiodensity after CL-316243 treatment and xenon-induced radiodensity enhancement (Fig. S2), we found only a moderate correlation. Given the relatively small increase in tissue radiodensity observed in the CT scans after CL-316243 treatment, the absence of correlation is not surprising. Similarly, we found even a lower correlation between tissue radiodensity enhancement after CL-316243 treatment and tissue glucose uptake. The latter, indeed, was also relatively small and had a large variability across mice.

The US studies qualitatively confirmed the increase in tissue vascularization after CL-316243 treatment detected using XECT scans, as well as the norepinephrine-induced increase in tissue perfusion observed before and after CL-316243 treatment. Unfortunately, quantitative measurements of tissue blood volume and blood flow were not always reliable, given the low perfusion of this tissue, even after CL-316243 treatment. Also, given the 2D nature of the scan and the observed heterogeneity of the browning process in inguinal fat, co-registration of post CL-316243 images with pre-CL-316243 images made the comparison even more challenging.

The present study has four major limitations. First, as comparisons were made in the same animals, before and after CL-316243 treatment, the increase in animal age and size that occurred during the treatment, which was clearly visible in all images, may have offset some of the changes induced in inguinal fat by CL-316243, and perhaps also contributed to the high variability observed across animals. A second limitation was the inability to exactly identify the same area in the 2D ultrasound scans which, along the heterogeneity of the tissue, made the comparison between pre-CL-316243 and post CL-316243 scans done with US less reliable. Third, while the primary goal of this study was to assess whether clinically used and widely available imaging modalities (PET/CT/US) could detect morphological and functional changes associated with the browning process in vivo, quantification of tissue oxidative metabolism or thermogenic activity would have been a more relevant outcome. However, measuring tissue oxidative metabolism and temperature in vivo in the inguinal depot of mice presents significant challenges. Oxygen consumption in beige fat could in principle be measured by using PET with ^15^O gas. This technique has already been used to measure the oxidative metabolism of the major BAT depot in humans^37–39^. However, because of the small half-life of this isotope, these measurements require special infrastructure (the presence of an on-site cyclotron, breathing and scavenging systems, etc.). Additionally, the reduced volume of inguinal fat and the observed heterogeneity of the browning process in this tissue, coupled with the limited resolution of PET will make any quantification extremely challenging. MR thermometry and infrared thermometry have been used by us^34,40,41^ and others^42,43^ to assess thermogenic activity of the main BAT depot in vivo. However, decoupling the increase in thermogenic activity of the inguinal depot from the much larger increase in thermogenic activity of BAT will be impossible, especially considering that in anesthetized mice thermogenic activity in BAT alone can increase whole body temperature by several degrees Celsius^40^. Finally, no comparison was made with chronic cold exposure treatment. Chronic cold exposure and chronic injection with CL-316243 are both widely used to induce browning of inguinal fat. In this study we choose CL-316243 treatment as cold exposure in mice requires special housing infrastructures which were not available in our facility. Nonetheless, while the degree of browning may be different between chronic cold exposure and chronic CL-316243 treatment, we are not aware of studies showing differences in the type of brown tissue formed under the two conditions.

In conclusion, CT, and xenon-enhanced CT scans were able to detect both changes in tissue water content as well as changes in tissue vascularization and perfusion induced by the browning process and norepinephrine stimulation, while no statistically significant increase in glucose uptake was observed in PET scans. Browning of inguinal fat was seen to be highly heterogeneous, with regions adjacent to the external iliac artery undergoing a more remarkable browning than more distal regions. Xenon enhanced CT scans acquired after norepinephrine stimulation of the tissue, before CL-316243 treatment, also showed a heterogenous radiodensity enhancement of the inguinal region, with the highest enhancement observed in those regions that showed an increase in tissue radiodensity post CL-316243 treatment. Given the possible role of sympathetic system dysfunction in obesity, these findings deserve more attention in future studies. The concurrent radiodensity increase observed after CL-316243 treatment in both xenon-enhanced and non-enhanced CT scans acquired before NE stimulation indicates that the increase in radiodensity is due to both an increase in cell water and protein content (originating from the conversion of unilocular adipocytes into UCP1-positive multilocular adipocytes) as well as to an increase in tissue perfusion. The increase in tissue cell water content and tissue perfusion, but the lack of a concurrent increase in tissue glucose uptake or vasculature reactivity to norepinephrine after CL-316243 treatment is interesting and consistent with previous studies showing lack of increase in tissue oxidative metabolism after CL-316243 treatment.

## Methods

### Animal protocol

All animal procedures were performed under an animal protocol approved by the Institutional Animal Care and Use Committee (IACUC) at the University of North Carolina at Chapel Hill and complied with relevant guidelines and regulations. The methods reported here also adhere to the recommendations in the ARRIVE guidelines. A timeline of the procedures performed on the animals is shown in Fig. 1. A total of 21 C57BL/6 J mice ranging from 2 to 3 months old were used for these studies. Mice were housed at 24 °C and kept under a 12:12-h light–dark cycle with ad libitum access to chow and water. Each animal underwent two ^18^F-FDG-PET/CT scans, two DCEUS scans, and two XECT scans, one right before and one right after a 4-week-long treatment with CL-1316243. To avoid co-registration artifacts that would arise from moving the animal between the 3 imaging platforms, PET/CT XECT and DCEUS were all performed on separate days. At the end of the last imaging study, mice were not recovered from anaesthesia but were euthanized using an overdose of pentobarbital at a dose of 200 mg/kg. After euthanasia, inguinal fat tissue was excised and prepared for immunohistochemical analysis.

### ^18^F-FDG-PET/CT scans

For the ^18^F-FDG-PET/CT imaging study, all mice were fasted overnight for at least 10 h prior to the PET scan. The following morning animals were anesthetized with 70 mg/kg of pentobarbital. After reaching a surgical level of anaesthesia, tail vein catheters were placed for radiotracer injection. Mice were then injected subcutaneously with 1 mg/kg of norepinephrine (NE). After 5 min, ^18^F-FDG was administered via tail vein catheter at a dose of 14.8 ± 1.3 MBq in 100 μL saline. PET/CT scans were acquired by using a small animal PET/CT system (SuperArgus model, Sedecal, Madrid Spain). Specifically, a 20-min static PET acquisition was initiated in all mice after the acquisition of CT images and at 45-min post-injection of the ^18^F-FDG radiotracer. At the end of the PET/CT scan, the animals were recovered from anaesthesia and returned to their cage.

CT images were acquired by using an X-ray peak energy of 70 kV, a current of 300 µA, 360 projections, and a linear voxel size of 210 µm. CT images were reconstructed using a FeldKamp algorithm with a nominal resolution of 0.21 mm. The PET resolution was 1.0 mm in the centre of the FOV, and close to 2 mm in the inguinal region analysed here. PET images were reconstructed with a 3D-OSEM algorithms and with a pixel size of 0.37 × 0.37 × 0.77 mm3.

### Xenon enhanced CT scans

For the xenon enhanced CT scans, mice were first anesthetized  with an intraperitoneal injection of 70 mg/kg pentobarbital and then intubated. Mechanical ventilation was performed by using a custom-made mechanical ventilator, which controlled the rate (80 breaths/minute), the volume (~ 0.2 ml) and composition (30/70 O_2_/N_2_ volume ratio for the non-enhanced CT scans and 30/70 O_2_/Xe volume ratio for the xenon-enhanced CT scans) of the inhaled gas. After anaesthesia induction and intubation, the animals were scanned by using a micro-CT scanner, Quantum GX2 system (Perkin Elmer, Waltham, Massachusetts) using the following parameters: 70 kV of peak voltage, 114 µA of current, a voxel size of 90 µm, and 46.1 mm FOV for one bed acquisition. After the acquisition of the first non-enhanced CT images, xenon inhalation was started. CT images were then acquired again at the end of a seven-minute xenon inhalation. At the end of the second CT scan, xenon inhalation was stopped. Ten minutes after the last xenon inhalation, the animals were injected with norepinephrine at a dose of 1 mg/kg. Five minutes later, a second non-enhanced CT scan was started. At the end of this scan, xenon inhalation was restarted, and a fourth CT scan was acquired at the end of the second seven-minute xenon inhalation. After the acquisition of the last CT images, the animals were recovered from anaesthesia and returned to their cage.

### Dynamic contrast enhanced ultrasound scans

For the dynamic contrast enhanced ultrasound studies, the animals were first anesthetized using 70 mg/kg of pentobarbital. An intravenous catheter was placed on the lateral tail vein for the injection of microbubbles. Abdominal fur was removed using depilatory cream, and ultrasound coupling gel was placed between the skin and the 18 MHz transducer (Vevo-2100, FUJIFILM Visual Sonics Inc., Bothell, WA). Consistent body temperature and respiration rate were maintained throughout the procedure. Following the identification of the inguinal lymph node using B-mode imaging, non-targeted microbubbles (Vevo Micro Marker FUJIFILM Visual Sonics Inc., Bothell, WA) were injected using a mechanical pump as a single bolus of 100 µL solution of 2 × 10^8^ $\mathrm{MB}/\mathrm{mL}$. Ten seconds later, contrast mode imaging was initiated. After ten minutes from the last microbubble injection, norepinephrine was administered subcutaneously at a dose of 1 mg/kg. Five minutes after norepinephrine injection, mice received a second injection of microbubbles at the same dose listed above and dynamic contrast ultrasound images were collected again. At the end of the imaging study, the animals were recovered from anesthesia and returned to their cage.

### CL-316243 treatment

After the acquisition of the first set of images, the animals began a 4-week-long treatment of 1 mg/kg intraperitoneal daily injections of CL-316243 (C5976, Sigma-Aldrich). Following the treatment, the animals underwent a second set of imaging experiments. As the entire imaging protocol took about 10 days, animals continued to receive additional CL-316243 injections during the days they were not imaged.

### Immunohistochemical analysis

Right after euthanasia the inguinal fat depots were excised and fixed in 4% paraformaldehyde at 4 °C for at least 24 h before storage in ethanol until tissues were paraffin-embedded for histological sectioning. Blocks were then sectioned into 4 μm slices, dewaxed, and rehydrated. Cyto-Q Background Buster (NB306; In-novex) was used for the blocking procedure, followed by UCP1 primary antibody incubation at room temperature (1:1000; catalogue no. ab10983; Abcam). A secondary antibody incubation was performed with biotinylated goat anti-rabbit (1:1000, BA100, Vector) and detection performed with Vectastain Elite ABC complex (Vector). Stained slides were scanned at 20× magnification and visualized using an optical microscope and later evaluated digitally using OlyVIA software (Olympus)*.*

### Imaging analysis

Calibration of CT values were performed for the pre and post CL-316243 treatment scans by using a water/air phantom. After calibration, quantification of mean tissue radiodensity was done by using ITK-snap and Horos by two independent investigators. For the analysis, volumes of interest (VOIs) were carefully drawn in the inguinal fat depot, around the external iliac artery in the post-CL-316243 scans. The same VOIs were identified and selected in the post-CL-316243 scans. Final tissue radiodensity values were then obtained by averaging values obtained by the two investigators.

For the ^18^F-FDG-PET analysis, two independent investigators drew volumes of interest (VOIs) adjacent to the external iliac artery in the left and right inguinal fat depot of post CL-316243 images using Horos and PMOD. Similar VOIs were then selected in the pre-CL-316243 scans. Left and right inguinal uptake values were independently averaged by the two investigators and a single SUV value was then derived from these two values. To account for inaccuracies due to catheter residue and as a control for plasma availability, average SUV values in the inguinal region were then divided by SUV values of VOIs selected in the triceps region to obtain standardized uptake value ratios (SUVR).

For the CEUS (Contrast Enhanced Ultrasound) analysis, regions of interest (ROIs) were manually selected within the inguinal fat depot, near the inguinal lymphnode. The average signal intensity within the ROI was automatically measured for each frame, after the burst of ultrasound waves, and modeled as *S(t)* = *A* × (1 − *e*^*−βt*^) + C, where *A* is the plateau intensity, proportional to the microvasculature blood volume, and *β* is the initial slope of the replenishment intensity time curve, proportional to the microvasculature flow velocity^31,32^. Because a measure of tissue blood flow in cm^2^/s could be obtained only if the beam elevation of the transducer and the microvasculature cross sectional area were known^31^, here, in absence of this information, the A and β parameters were used only to provide a qualitative measure of tissue blood volume and tissue blood flow.

### Statistical analysis

Because of the high heterogeneity observed in the inguinal region, two independent observers were asked to independently analyze the data. A Kendall’s tau-b test and the associated p-value was computed to assess cross correlation between the values observed by the two observers. SUV and HU (Hounsfield Units) values obtained by the two observers were then averaged to reduce systematic error. Comparison between radiodensity and SUV values obtained after each treatment were made by using a two-sided paired t-test. Mean values, standard deviations, and p-values for the observed enhancements are reported in the Supplementary Material. To assess differences in SUV and radiodensity enhancement between males and females we used a heteroscedastic t-test, while also computing the Cohen’s d value to assess effect size.

### Supplementary Information

Supplementary Information.

## Data Availability

The datasets generated during and/or analyzed during the current study are available from the corresponding author on reasonable request.
