# Supplementary material for: In-vivo detection of white adipose tissue browning: a multimodality imaging approach
Source: Sci Rep. 2023 Sep 19;13:15485. doi: 10.1038/s41598-023-42537-9 (PMC10509182; doi:10.1038/s41598-023-42537-9)
Supplement: Supplementary file 1 — Supplementary Information. [file 41598_2023_42537_MOESM1_ESM.pdf]

Supplementary Material for

In-Vivo Detection of White Adipose Tissue Browning: A Multimodality Imaging Approach

Leah R Holmes[1,2], John C Garside[1,2], Jonathan Frank[2], Eric Livingston[2], Jonas Snyder[1,2], Nada Abu Khalaf[1,2], Hong Yuan[2], Rosa T Branca[1,2*]

[1] Department of Physics and Astronomy, University of North Carolina at Chapel Hill, Chapel Hill, NC 27599
[2] Biomedical Research Imaging Center, University of North Carolina at Chape Hill, Chapel Hill, NC 27599

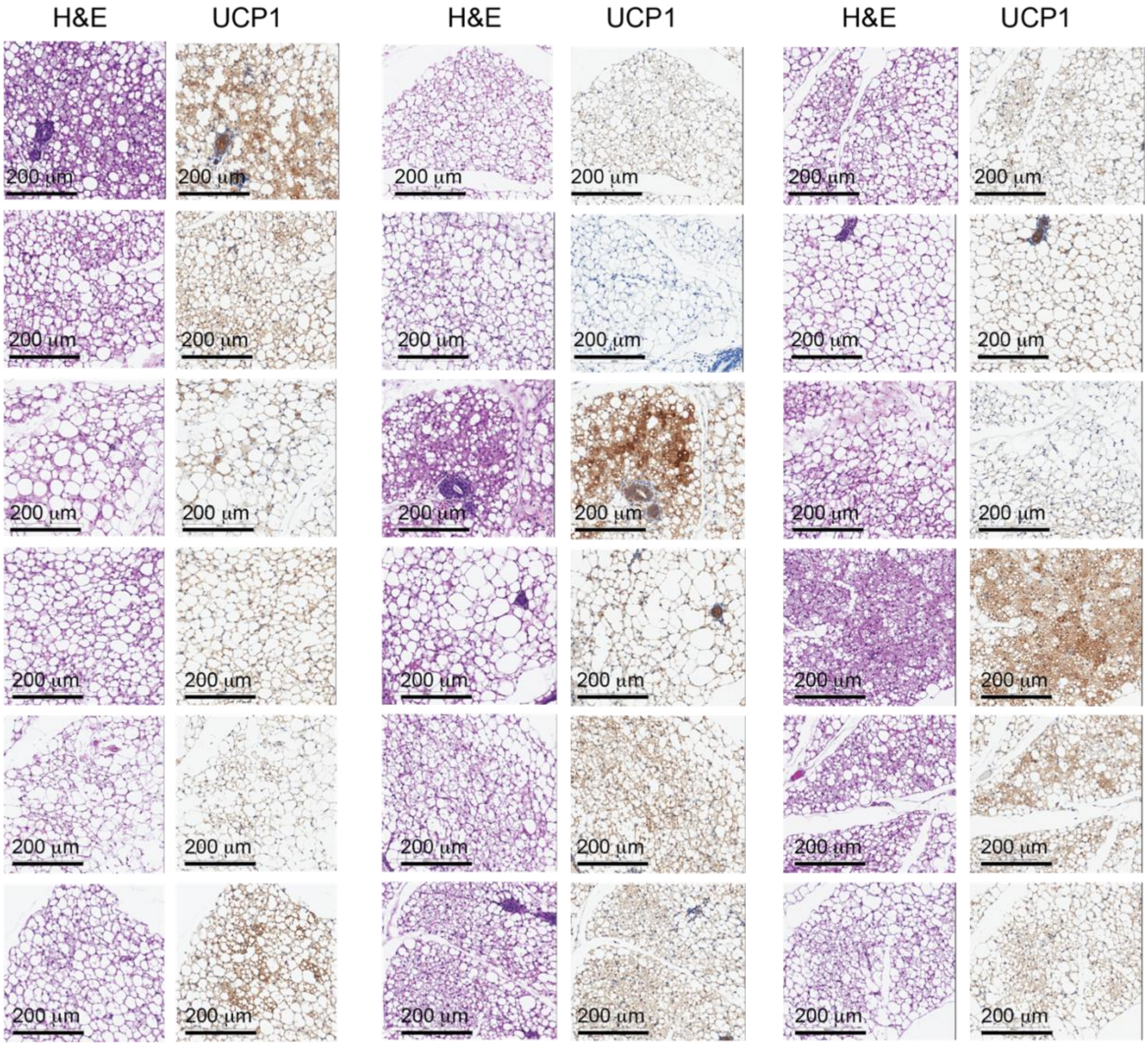

Figure S1: Examples of hematoxylin and eosin (H&E) and UCP1-stained images of inguinal BAT obtained from this study from different animals showing different degrees of tissue browning. The tissue displayed a high degree of heterogeneity at the microscopic level, in most cases with clusters of multilocular UCP1-positive adipocytes next to unilocular white adipocytes.

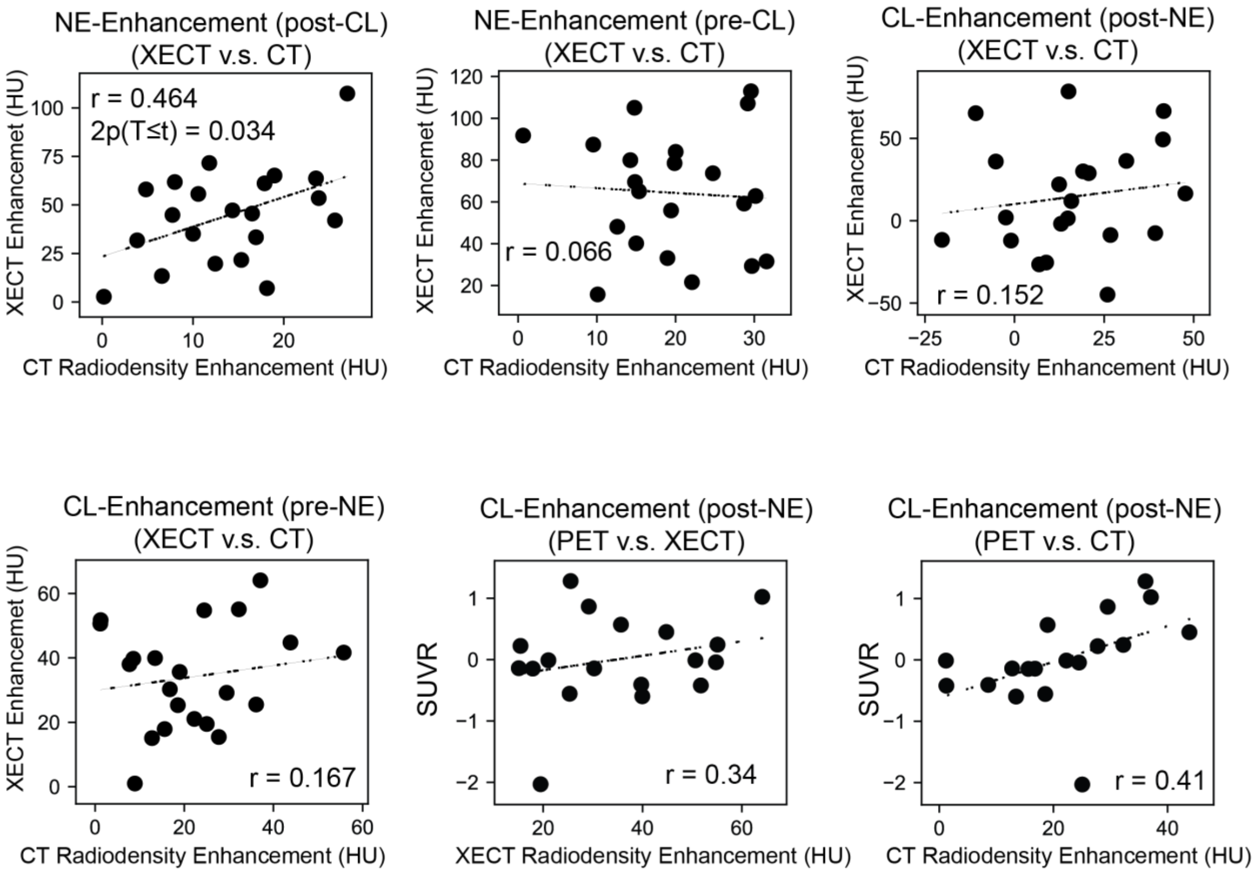

Figure S2. Correlation plots showing a moderate correlation (Pearson's r) between tissue radiodensity enhancement observed in CT scans after NE injection and xenon-induced radiodensity enhancement observed in the same tissue after NE injection. This correlation was present after CL-316243 (post-CL) treatment but not before CL-316243 treatment (pre-CL)). A moderate (Pearson's r =0.41) but not statistically significant ($p > 0.05$) correlation was also observed between the increase in tissue radiodensity observed in CT scans after CL-316243 treatment, and the increase in tissue glucose uptake observed after CL-316243 treatment.

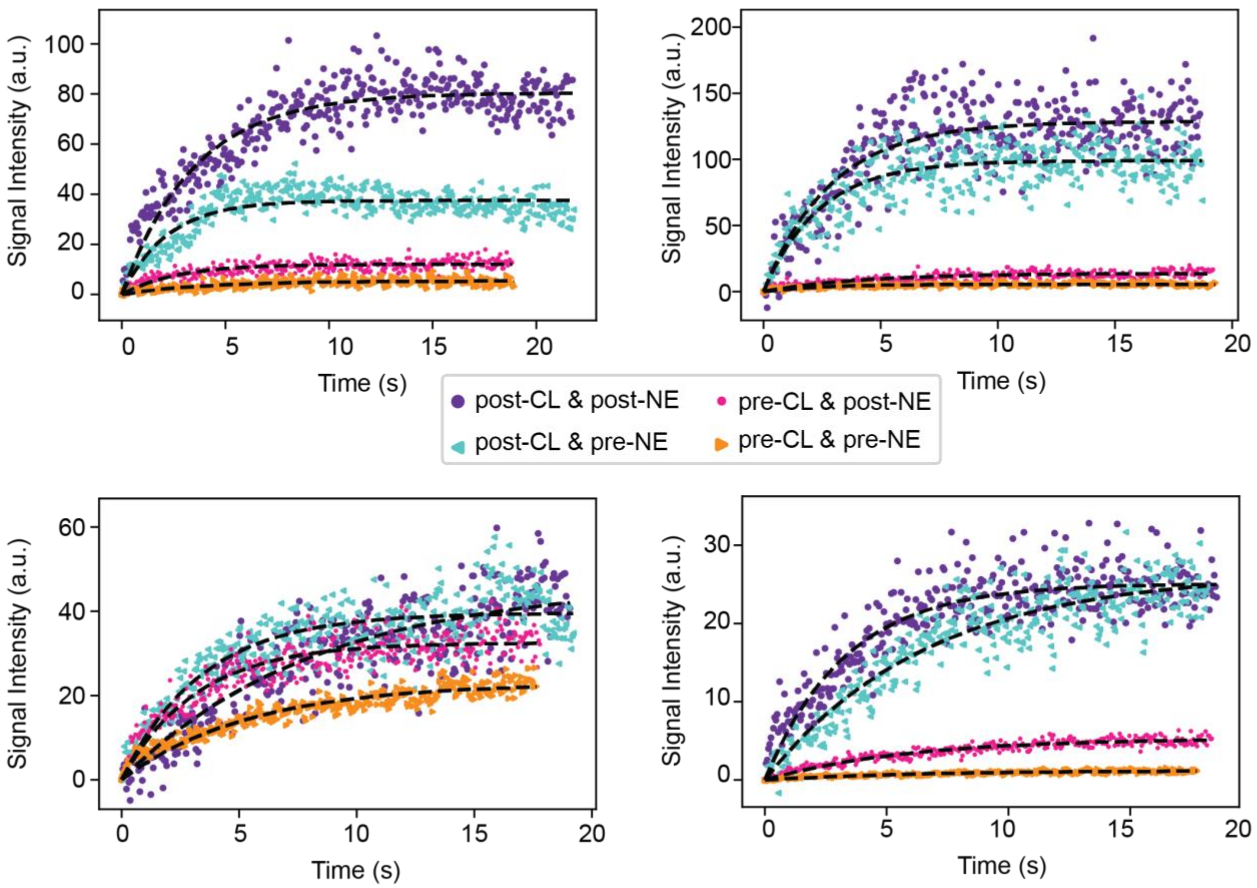

Figure S3: Additional examples of dynamic ultrasound data collected as part of this study. Signal enhancement after norepinephrine injection was observed in most but not all mice, more after CL-316243 treatment (post-CL) than before CL-316243 treatment (pre-CL). Fitting values for all dynamics US data is provided in Table 5

Inguinal SUV values	pre-CL SUV			post-CL SUV			Uptake Enhancement (SUV)
	**Reader 1**	**Reader 2**	**Avg**	**Reader 1**	**Reader 2**	**Avg**	**Avg**
**M05**	0.2	0.26	0.23	0.35	0.38	0.36	0.14
**M07**	0.21	0.19	0.15	0.32	0.33	0.33	0.18
**M22**	0.33	0.31	0.33	0.17	0.19	0.18	-0.15
**M23**	0.4	0.35	0.38	0.24	0.27	0.25	-0.12
**M24**	0.31	0.31	0.31	0.2	0.38	0.29	-0.02
**M26**	0.23	0.18	0.23	0.14	0.15	0.15	-0.08
**M27**	0.15	0.17	0.16	0.15	0.2	0.18	0.02
**M29**	0.36	0.36	0.36	0.44	0.48	0.46	0.1
**M30**	0.45	0.47	0.4	0.27	0.28	0.27	-0.13
**M31**	0.29	0.21	0.25	0.46	0.62	0.54	0.29
**M32**	0.16	0.15	0.15	0.23	0.24	0.23	0.08
**M33**	0.79	1.04	0.85	0.22	0.24	0.23	-0.62
**M34**	0.38	0.47	0.42	0.75	0.7	0.72	0.3
**M36**	0.43	0.46	0.44	0.62	0.92	0.77	0.33
**M41**	0.28	0.26	0.26	0.33	0.41	0.37	0.11
**M42**	0.26	0.28	0.27	0.27	0.4	0.34	0.06
**M51**	0.2	0.17	0.19	0.28	0.33	0.3	0.12
**M52**	0.5	0.58	0.55	0.35	0.35	0.35	-0.2
**M60**	0.23	0.3	0.18	0.08	0.15	0.11	-0.07

Table 1: Average standardized uptake values, as measured from the left and right inguinal depot, by the two readers on independently drawn volumes of interest.

Triceps SUV values	pre-CL Uptake (SUV)			post-CL Uptake (SUV)			Enhancement Uptake (SUV)
	Reader 1	Reader 2	Avg	Reader 1	Reader 2	Avg	Avg
**M05**	0.23	0.25	0.24	0.24	0.24	0.24	0
**M07**	0.11	0.13	0.12	0.21	0.19	0.2	0.08
**M22**	0.2	0.22	0.21	0.18	0.14	0.16	-0.05
**M23**	0.28	0.24	0.26	0.19	0.17	0.18	-0.08
**M24**	0.22	0.25	0.24	0.3	0.21	0.26	0.02
**M26**	0.19	0.24	0.21	0.18	0.19	0.18	-0.03
**M27**	0.16	0.19	0.17	0.24	0.22	0.23	0.05
**M29**	0.23	0.28	0.26	0.36	0.36	0.36	0.11
**M30**	0.26	0.23	0.21	0.2	0.17	0.19	-0.02
**M31**	0.17	0.22	0.19	0.22	0.2	0.21	0.02
**M32**	0.25	0.27	0.26	0.29	0.27	0.28	0.02
**M33**	0.19	0.33	0.23	0.15	0.16	0.16	-0.08
**M34**	0.27	0.33	0.28	0.28	0.31	0.3	0.01
**M36**	0.25	0.27	0.26	0.27	0.33	0.3	0.04
**M41**	0.16	0.18	0.14	0.22	0.19	0.2	0.07
**M42**	0.26	0.2	0.23	0.23	0.18	0.2	-0.02
**M51**	0.16	0.13	0.15	0.23	0.25	0.24	0.1
**M52**	0.23	0.23	0.23	0.19	0.2	0.19	-0.04
**M60**	0.19	0.19	0.17	0.15	0.14	0.14	-0.03

Table 2: Average standardized uptake values as measured from the left and right triceps by the two readers on independently drawn volumes of interest.

Inguinal SUVR	Pre-CL Uptake (SUV)	Post-CL Uptake (SUV)
**M05**	0.95	1.52
**M07**	1.66	1.62
**M22**	1.53	1.11
**M23**	1.45	1.43
**M24**	1.31	1.11
**M26**	0.94	0.8
**M27**	0.91	0.77
**M29**	1.41	1.27
**M30**	1.87	1.46
**M31**	1.29	2.57
**M32**	0.59	0.83
**M33**	3.52	1.49
**M34**	1.42	2.45
**M36**	1.69	2.55
**M41**	1.6	1.82
**M42**	1.19	1.64
**M51**	1.28	1.26
**M52**	2.34	1.79
**M60**	1.41	0.81

Table 3: Standardized uptake value ratios measured by dividing the average inguinal SUV values obtained by the two readers, by the average muscle SUV values as measured by the two readers.

	Reader 1				Reader 2			
**Mouse ID**	**pre-CL pre-NE (HU)**	**pre-CL post-NE (HU)**	**post-CL pre-NE (HU)**	**post-CL post-NE (HU)**	**pre-CL pre-NE (HU)**	**pre-CL post-NE (HU)**	**post-CL pre-NE (HU)**	**post-CL post-NE (HU)**
M05	-130	-118	-104	-100	-147	-120	-135	-108
M07	-121	-121	-92	-70	-126	-125	-106	-81
M12	-136	-113	-80	-86	-152	-137	-97	-81
M22	-133	-114	-124	-119	-141	-116	-147	-132
M23	-119	-101	-122	-112	-144	-103	-138	-133
M26	-158	-130	-135	-111	-163	-134	-152	-122
M27	-152	-132	-137	-123	-161	-152	-144	-143
M29	-143	-126	-136	-114	-173	-171	-154	-129
M30	-136	-118	-119	-116	-133	-122	-132	-128
M31	-129	-112	-70	-66	-131	-117	-116	-84
M32	-142	-122	-102	-87	-158	-138	-134	-111
M33	-134	-106	-105	-90	-140	-105	-119	-82
M34	-133	-123	-85	-75	-143	-127	-117	-93
M36	-146	-115	-95	-90	-142	-113	-134	-106
M37	-134	-123	-127	-126	-142	-133	-131	-132
M41	-123	-107	-79	-71	-121	-108	-109	-92
M42	-131	-99	-68	-78	-133	-106	-108	-85
M51	-147	-126	-110	-113	-159	-140	-151	-126
M52	-147	-117	-108	-104	-162	-133	-164	-132
M53	-134	-105	-113	-105	-152	-130	-157	-141
M60	-128	-105	-104	-98	-140	-132	-137	-114

Table 4: Inguinal radiodensity in Hounsfield units as measured by the two readers in non-enhanced CT images. Values are averaged values from the right and left inguinal depot.

	Reader 1				Reader 2			
**Mouse ID**	**pre-CL pre-NE**	**pre-CL post-NE**	**post-CL pre-NE**	**post-CL post-NE**	**pre-CL pre-NE**	**pre-CL post-NE**	**post-CL pre-NE**	**post-CL post-NE**
**M05**	-112	-16	-73	-60	-137	-122	-104	-75
**M07**	-121	-36	-63	-1	-123	-24	-71	-26
**M12**	-121	-81	-100	-26	-143	-116	-80	-38
**M22**	-109	-91	-49	-14	-110	-85	-67	-31
**M23**	-100	1	-39	13	-110	3	-69	-32
**M26**	-134	-87	-96	-15	-146	-75	-123	10
**M27**	-130	-46	-101	-61	-142	-16	-135	-51
**M29**	-103	-11	-105	-46	-163	-80	-131	-63
**M30**	-101	-32	-63	-26	-105	-35	-64	-37
**M31**	-92	-50	-48	-49	-91	-53	-84	-68
**M32**	-122	-30	-63	-3	-142	-66	-91	-21
**M33**	-76	-32	-49	4	-81	-61	-69	-38
**M34**	-106	-37	-33	-10	-126	-98	-71	-27
**M36**	-96	-31	-65	-20	-122	-61	-94	-49
**M37**	-121	-103	-127	-118	-136	-123	-128	-131
**M41**	-102	-22	-71	-50	-102	-23	-103	-84
**M42**	-105	-74	-52	-39	-118	-91	-82	-68
**M51**	-110	-13	-86	-15	-148	-88	-130	-90
**M52**	-111	8	-76	-11	-148	-40	-132	-74
**M53**	-91	-13	-61	14	-124	-55	-78	-11
**M60**	-107	-21	-62	-8	-133	-88	-98	-57

Table 5: Inguinal radiodensity values as measured in xenon-enhanced CT scans by the two readers.

CT	Pre-NE PostCL-Pre CL	Post-NE PostCL-Pre CL	Pre-CL PostNE- PreNE	PostCL PostNE-PreNE
**Mean Increase (HU)**	22	16	20	14
**Standard Deviation of Increase (HU)**	14	18	8	7
**p-value increase**	< 0.0001	< 0.001	< 0.0001	< 0.0001
**Mean Increase Male (HU)**	12	4	21	13
**Standard Deviation of Increase in Male (HU)**	7	15	7	8
**p value increase**	0.0015	0.5	< 0.001	< 0.001
**Mean Increase Female (HU)**	30	26	18	14
**Standard Deviation of Increase in Female (HU)**	13	15	9	8
**p value increase**	< 0.0001	< 0.0001	< 0.0001	< 0.0001
**p value for difference in increase between male and female (2-sides heteroscedastic t-test)**	< 0.0001	0.003	> 0.05	> 0.05
**Cohen's d**	-1.27	-1.22	0.4	-0.15

XECT-CT	PreCL & PreNE	PostCL & PreNE	PreCL & PostNE	PostCL & PreNE
**Mean Increase (HU)**	24	36	69	67
**Standard Deviation of Increase (HU)**	12	22	29	33
**p-value increase**	< 0.0001	< 0.0001	< 0.0001	< 0.0001
**Mean Increase Male (HU)**	26	47	84	92
**Standard Deviation of Increase in Male (HU)**	5	23	28	22
**p value increase**	< 0.0001	0.0003	< 0.0001	< 0.0001
**Mean Increase Female (HU)**	22	28	57	48
**Standard Deviation of Increase in Female (HU)**	15	18	25	27
**p value increase**	0.0005	0.0002	< 0.0001	< 0.0001
**p value for difference in increase between male and female (2-sides heteroscedastic t-test)**	> 0.05	> 0.05	0.04	0.0005
**Cohen's d**	0.35	0.85	0.92	1.34

XECT	Pre-NE PostCL-Pre CL	Post-NE PostCL-Pre CL	Pre-CL PostNE-PreNE	PostCL PostNE-PreNE
**Mean Increase (HU)**	34	15	64	45
**Standard Deviation of Increase (HU)**	16	33	29	25
**p-value increase**	< 0.0001	0.056	< 0.0001	< 0.0001
**Mean Increase Male (HU)**	32	12	79	59
**Standard Deviation of Increase in Male (HU)**	14	39	28	22
**p value increase**	< 0.0001	> 0.05	< 0.0001	< 0.0001
**Mean Increase Female (HU)**	36	17	53	34
**Standard Deviation of Increase in Female (HU)**	18	30	24	21
**p value increase**	< 0.0001	> 0.05	< 0.0001	0.0002
**p value for difference in increase between male and female (2-sides heteroscedastic t-test)**	> 0.005	> 0.005	0.04	0.019
**Cohen's d**	-0.2	-0.13	0.9	1

Table 6: Mean increase and standard deviation of the enhancement observed in all mice as reported in the plots of Figure 5. P-values and Cohen's d values were also used to assess statistically significant differences in the enhancement observed between male and female.

	Post-CL & Post-NE. SE(β)						Post-CL & Pre-NE					
	**A**	β	**SE(A)**	**SE(β)**	$\chi2$	**S**	**A**	β	**SE(A)**	**SE(β)**	$\chi2$	**S**
**M1**	36.23	0.07	1.85	0.01	32.27	0.03	7.19	0.54	0.15	0.06	74.66	0.02
**M13**	9.07	0.17	0.29	0.01	103.23	0.02	4.61	0.41	0.09	0.04	256.77	0.01
**M14**	36.92	0.3	0.43	0.01	15.11	0.05	25.01	0.32	0.33	0.02	32.37	0.04
**M15**	2.21	0.07	0.12	0.01	> 500	0	1.59	0.09	0.07	0.01	> 500	0
**M5**	190.74	0.17	4.4	0.01	0.52	0.26	167.02	0.32	2.24	0.02	0.52	0.26
**M7**	215.08	0.22	2.56	0.01	0.75	0.22	61.25	0.32	0.72	0.02	4.96	0.08
**M12**	67.39	0.26	1.22	0.01	3.83	0.12	34.33	0.11	1.81	0.01	25.19	0.05
**M22**	46.57	0.12	1.57	0.01	9.07	0.06	39.6	0.29	0.45	0.01	13.91	0.05
**M23**	25.09	0.3	0.24	0.01	48.43	0.03	26.53	0.15	0.51	0.01	51.98	0.03
**M31**	162.17	0.48	2.02	0.03	0.42	0.28	127.82	0.32	1.62	0.02	0.97	0.19
**M32**	171.7	0.23	2.68	0.01	0.57	0.24	167.33	0.33	1.82	0.02	0.71	0.21
**M34**	103.49	0.15	2.42	0.01	2.3	0.13	59.25	0.24	1.03	0.01	3.85	0.1
**M36**	22.51	0.33	0.28	0.02	29.11	0.03	15.59	0.48	0.15	0.03	71.95	0.02
**M37**	83.25	0.23	1.21	0.01	3.13	0.11	49.49	0.15	2.39	0.02	2.33	0.12
**M41**	128.6	0.35	1.6	0.02	0.9	0.2	98.98	0.42	1.09	0.02	1.6	0.15
**M42**	81.52	0.4	1.89	0.05	0.56	0.25	64.32	0.85	0.77	0.08	2.21	0.13
**M60**	28.74	0.29	1.14	0.05	2.27	0.12	28.04	0.25	0.51	0.02	14.75	0.05
**M51**	80.43	0.29	0.63	0.01	6.03	0.06	37.51	0.46	0.32	0.02	15.53	0.04
**M31-**	116.88	5.24	0.8	0.71	1.64	0.14	55.59	0.42	0.38	0.01	13.06	0.05
**M7-BAT**	200.15	4.79	2.24	1.04	0.21	0.38	56	0.15	1.34	0.01	5.95	0.07
	**Pre-CL & Post-NE**						**Pre-CL & Pre-NE**					
	**A**	β	**SE(A)**	**SE(β)**	$\chi2$	**S**	**A**	β	**SE(A)**	**SE(β)**	$\chi2$	**S**
**M5**	42.43	0.35	0.67	0.02	5.4	0.08	65.44	0.41	0.77	0.02	3.41	0.1
**M7**	18.04	0.34	0.23	0.02	56.98	0.02	6.97	0.23	0.12	0.01	496.5	0.01
**M22**	22.91	0.18	0.28	0.01	108.28	0.02	32.52	0.31	0.28	0.01	34.86	0.03
**M23**	1.2	0.17	0.03	0.01	>500	0	5.34	0.17	0.07	0.01	>500	0
**M31**	47.17	0.12	1.89	0.01	5.41	0.08	66.28	0.17	1.22	0.01	5.18	0.08
**M32**	11.9	0.34	0.19	0.03	60.97	0.02	13.35	0.21	0.25	0.01	69.68	0.02
**M34**	61.47	0.5	0.73	0.03	3.02	0.11	56.72	0.25	0.98	0.02	4.02	0.1
**M36**	27.76	0.26	0.41	0.02	15.76	0.05	50.57	0.24	0.73	0.01	5.76	0.07
**M37**	3.89	0.29	0.05	0.02	> 500	0.01	2.97	0.03	1.23	0.01	> 500	0
**M41**	5.59	0.38	0.08	0.03	296.79	0.01	13.8	0.24	0.24	0.01	66.76	0.02
**M42**	4.93	0.27	0.07	0.01	708.71	0.01	10.95	0.26	0.13	0.01	183.7	0.01
**M60**	6.74	0.46	0.06	0.02	502.41	0.01	10.1	0.24	0.12	0.01	248.8	0.01
**M51**	5.39	0.25	0.13	0.02	223.04	0.01	12.07	0.38	0.14	0.02	102.5	0.02

Table 7: A and β parameters as obtained by fitting ultrasound dynamics time curves. The table provides both the chi-squared value for the fit $\chi2$, as well as the standard error of the regression S.

Kendall's tau rank test.

Both FDG-PET and CT images were analyzed by two independent investigators. A Kendall's tau-b rank test was then computed to assess differences in the values obtained by the two investigators for the same animal using independently drawn VOIs. This test showed a strong and significant positive correlation between the two readers standardized uptake values in the inguinal (τ_b=*0.53, p < 0.0001*) and muscle (τ_b=*0.78, p < 0.0001*) region, as well as for the CT (τ_b=*0.60, p< 0.0001*) and XECT (τ_b=*0.66, 2PT≤t = 0.0001*) radiodensity values measured by the two investigators on the same animal from the independently drawn VOIs.
